# Innovative Approaches to Medical Rehabilitation: Regeneration, Immune Training, Homeostasis, and Microbiome Synergy

**DOI:** 10.3390/ijms26178687

**Published:** 2025-09-06

**Authors:** Enrico Garaci, Matteo Antonio Russo, Marilena Pariano, Matteo Puccetti, Consuelo Fabi, Sarah Balucchi, Marina Maria Bellet, Maurizio Ricci, Massimo Fini, Luigina Romani

**Affiliations:** 1 San Raffaele Research Center, 67039 Sulmona, Italy; garenco@icloud.com; 2 IRCCS San Raffaele, 00163 Rome, Italy; matteoantoniorusso44@gmail.com (M.A.R.); massimo.fini@sanraffaele.it (M.F.); 3 Department of Medicine and Surgery, University of Perugia, 06123 Perugia, Italy; marilena.pariano@gmail.com (M.P.); consuelofabi93@gmail.com (C.F.); s.balucchi97@gmail.com (S.B.); marinamaria.bellet@unipg.it (M.M.B.); 4 Department of Pharmaceutical Sciences, University of Perugia, 06123 Perugia, Italy; matteo.puccetti@unipg.it (M.P.); maurizio.ricci@unipg.it (M.R.)

**Keywords:** regenerative medicine, systemic homeostasis, immunological training, microbiome modulation, holistic rehabilitation

## Abstract

This article explores an integrative framework for medical rehabilitation that combines regenerative medicine, systemic homeostasis, and microbiome modulation to optimize recovery and long-term health. Moving beyond conventional rehabilitation approaches focused on symptomatic recovery, this multidimensional paradigm emphasizes cellular repair, physiological balance, and microbial health as interdependent pillars of effective recovery. The framework leverages advancements in stem cell therapy, immune system modulation, and microbiota-targeted interventions to address both immediate functional restoration and long-term systemic resilience. By highlighting the synergistic interplay between these components, this article provides actionable insights into transforming medical rehabilitation into a proactive and holistic endeavor, paving the way for enhanced therapeutic outcomes and sustained patient well-being.

## 1. Introduction

The field of medical rehabilitation is undergoing a transformative evolution, driven by advancements in science and technology that challenge traditional paradigms [1,2]. Historically, rehabilitation has focused on restoring physical functionality following injury or illness, emphasizing therapies aimed at the symptomatic recovery of motor, sensory, or cognitive impairments. However, this conventional scope is expanding to encompass a broader, more integrative framework that addresses the body’s systemic capacity to heal, adapt, and thrive [3]. This paradigm shift combines regenerative medicine, homeostatic interventions, and immunological training into a cohesive strategy that seeks not only to recover lost functionality but also to enhance the body’s intrinsic mechanisms for healing and long-term maintenance [4,5].

At the core of this approach lies the recognition that recovery is not merely a matter of repairing isolated damage but a process involving the intricate interplay of biological systems. Regenerative medicine offers tools to repair and replace damaged tissues at different levels, employing innovations such as stem cells and tissue engineering [6,7,8]. Homeostatic interventions, in turn, stabilize the internal physiological environment, ensuring that systemic balance—critical for health and recovery—is restored and sustained. Immunological training refines the body’s defense and repair capabilities, enabling precise and effective responses to injury or disease [9,10]. Together, these approaches create a dynamic framework for addressing the complexities of recovery in diverse clinical scenarios.

Emerging research underscores the pivotal role of the human microbiome—a vast ecosystem of microorganisms residing within the body—in maintaining homeostasis and influencing overall health [11]. The gut microbiota, in particular, acts as a central regulator of immune function, metabolic processes, and neurophysiological health [12,13,14]. Beyond digestion, it influences inflammation, hormonal regulation, and even mood and cognition. The profound connection between microbiome health and systemic rehabilitation has brought microbiome modulation—through probiotics, prebiotics, and postbiotics—into the spotlight as an essential component of modern therapeutic strategies [15].

The convergence of these fields signals a new frontier in medical rehabilitation, where recovery is viewed as a multidimensional process involving cellular repair, systemic balance, and microbial health. This integrated approach offers the potential to not only restore lost functionality but also to preemptively enhance resilience and adaptability, setting the stage for a more holistic and comprehensive vision of health care. By leveraging these advancements, medical science is poised to redefine rehabilitation as a proactive endeavor that fosters systemic restoration, resilience, and lifelong well-being.

## 2. A Holistic Framework: From Individual Interventions to Systemic Synergy

The intersection of regenerative medicine, homeostatic interventions, immunological training, and microbiome modulation offers a holistic approach to medical rehabilitation. These components are interconnected, with each contributing to a unified goal of systemic restoration and resilience. The paradigm of medical rehabilitation is, indeed, increasingly characterized by a systemic and integrated approach that combines regenerative medicine, homeostatic interventions, immunological training, and microbiome modulation [16]. Overall, the integration of regenerative therapies, homeostatic interventions, immunological training, and microbiome modulation represents a holistic approach to rehabilitation [1,3]. These components are deeply interconnected, with each reinforcing the others to foster functional recovery and achieve systemic resilience in that (a) Regenerative therapies provide the structural basis for recovery, creating a platform for functional restoration; (b) Homeostatic interventions stabilize internal systems, ensuring a conducive environment for healing; (c) Immunological training refines defense mechanisms, preventing complications and optimizing repair; (d) Microbiome modulation supports these processes by enhancing immune regulation, reducing inflammation, and improving systemic health. This multidimensional approach shifts the focus from isolated treatments to comprehensive strategies that address the full spectrum of recovery needs, paving the way for more resilient and adaptive health outcomes [4]. Together, they contribute to a unified goal of health restoration, where the emphasis is on addressing both the immediate and long-term challenges of recovery. Box 1 schematically summarizes how a multidimensional approach offers a proactive vision for rehabilitation, addressing both immediate recovery needs and long-term health outcomes.

Box 1 **The multidimensional approach to medical rehabilitation.**

- *Regenerative Medicine: Building the Foundation*

At the core of the multidimensional framework for rehabilitation is regenerative medicine, a field focused on repairing and replacing damaged tissues and organs with innovative techniques. These techniques enable cellular rejuvenation and repair, offering new hope for conditions previously considered untreatable. From musculoskeletal injuries to neural damage, regenerative medicine serves as the cornerstone of recovery, creating a foundation upon which other therapeutic strategies can build [17,18,19,20,21].

- *Homeostatic Interventions: Stabilizing the System*

Effective rehabilitation requires more than repairing damaged tissues; it demands a stable physiological environment conducive to healing. Homeostatic interventions focus on restoring and maintaining internal equilibrium, addressing systemic imbalances that can hinder recovery. These interventions encompass metabolic regulation, hormonal balancing, and the correction of chronic dysregulation, creating an optimized environment for cellular repair and systemic resilience. By ensuring stability at the core of the body’s systems, homeostatic approaches provide the groundwork for sustained recovery and prevention of relapse [22,23,24,25]

- *Immunological Training: Refining Biological Responses*

The immune system plays a dual role in recovery, acting both as a driver of healing and a potential impediment when dysregulated. Immunological training harnesses the immune system’s power to balance pro-inflammatory and anti-inflammatory responses, ensuring optimal healing conditions. By modulating immune pathways, therapies can prevent chronic inflammation, stimulate angiogenesis, and promote tissue repair [26,27,28,29]. This shows how immunological interventions, from cytokine therapies to adoptive immune cell transfer, refine the body’s natural defenses to enhance recovery and reduce complications.

- *Microbiome Modulation: The Overlooked Partner*

The human microbiome is increasingly recognized as a critical factor in rehabilitation and systemic health. Particularly, the gut microbiota influences immune regulation, metabolic stability, and neurophysiological processes, all of which are central to recovery. Microbiome modulation through prebiotics, probiotics, and postbiotics supports these functions, offering a complementary approach to conventional rehabilitation strategies [11,30,31,32,33,34,35,36,37,38]. This underlies the concept of microbiome management as a therapeutic tool, emphasizing its potential to improve outcomes across diverse rehabilitation contexts.

- *Interconnected Mechanisms*

Rehabilitation is not merely the sum of isolated interventions but a dynamic interplay of interconnected systems. Regenerative medicine, homeostasis, immunity, and microbiome modulation do not function in silos; instead, they form a synergistic network that supports recovery at multiple levels [14,39,40]. These components interact, reinforcing one another to enhance outcomes.

## 3. Regenerative Medicine

At the heart of modern rehabilitation lies regenerative medicine, which focuses on repairing or replacing damaged tissues to restore functionality (Table 1). Innovations such as stem cell therapy, 3D bioprinting, and tissue engineering have revolutionized the field, offering the ability to rejuvenate tissues at a cellular level [21]. These techniques enable cellular rejuvenation and repair, offering new hope for conditions previously considered untreatable [17,18,19,20,21]. For instance, mesenchymal stem cells (MSCs), due to their self-renewal, pluripotency and immunomodulatory properties, have been shown to promote angiogenesis, reduce inflammation, and stimulate endogenous repair processes, making them a cornerstone of regenerative efforts [41,42]. The therapeutic effects of MSCs can be mediated through the release of bioactive molecules, including growth factors, cytokines, and extracellular vesicles, which play crucial roles in modulating the local cellular environment and exerting anti-inflammatory effects. MSCs can also interact with various immune cells, such as T cells, B cells, dendritic cells, and macrophages, modulating the immune response through both direct cell–cell interactions and the release of immunoregulatory molecules [42]. However, while stem cells have received significant attention, the manipulation of stem cell niches to boost regeneration is the subject of intense investigation, as signaling pathways, cell fate, and mobilization are influenced by the surrounding physical, biological, and chemical environment and the dynamic niche signals facilitate change in stem cell behavior, e.g., from quiescence to active tissue regeneration [43,44]. Moreover, the advent of biomaterials and scaffold-based approaches allows for the creation of bioengineered tissues that closely mimic natural structures [45,46]. These techniques are particularly relevant for musculoskeletal injuries, where precise anatomical reconstruction is critical. By addressing the structural damage underlying functional impairments, regenerative therapies provide a foundation for comprehensive recovery.

## 4. Homeostatic Interventions

Restoration of homeostasis—defined as the body’s ability to maintain stable internal conditions—is another crucial aspect of rehabilitation. Homeostatic interventions focus on stabilizing physiological systems such as metabolism, hormone regulation, and cardiovascular function to create an optimal environment for healing [60,61,62]. For example, metabolic recalibration through personalized nutritional strategies has demonstrated significant benefits in patients with chronic diseases, helping to reduce systemic inflammation and improve energy availability [23,24]. Similarly, hormonal interventions targeting thyroid or adrenal imbalances can enhance recovery outcomes by optimizing cellular repair and regeneration processes. These interventions not only address existing imbalances but also provide a preventative framework, reducing the possibility of recurrent health issues.

## 5. The Role of the Immune System in Rehabilitation and Tissue Regeneration

The immune system is a central orchestrator of rehabilitation and tissue regeneration, playing multifaceted roles in initiating, sustaining, and resolving healing processes. Through its ability to modulate inflammation, promote angiogenesis, stimulate repair mechanisms, resolve inflammation, and facilitate immunomodulation, the immune system integrates multiple biological pathways to support effective tissue regeneration. However, the immune system is a double-edged sword in recovery, capable of both facilitating and hindering the healing process [63]. For expanding the concept of medical rehabilitation to include immunological interventions, the following considerations may be taken into account:

- *The Immune System as a Driver of Healing*

The immune system initiates the healing process through inflammation, a natural response to injury or infection. In the acute phase, immune cells such as macrophages and neutrophils infiltrate the damaged tissue, releasing pro-inflammatory cytokines like interleukin-1 (IL-1) and tumor necrosis factor-alpha (TNF-α). These cytokines not only recruit additional immune cells to the site but also clear cellular debris and pathogens, laying the groundwork for subsequent repair. This inflammatory phase is indispensable for activating downstream processes such as angiogenesis, tissue remodeling, and stem cell activation. However, the challenge lies in ensuring that this phase is appropriately regulated to avoid chronic inflammation, which can lead to fibrosis, delayed healing, autoimmunity, or even cancer progression [64], making precise immune regulation critical [65].

- *The Immune System Enhances Angiogenesis*

Angiogenesis, the formation of new blood vessels, is a critical aspect of tissue repair that is closely regulated by the immune system. Immune cells such as macrophages and T-cells release vascular endothelial growth factor (VEGF) and platelet-derived growth factor, key signaling molecules that stimulate endothelial cell proliferation and migration. This vascularization ensures that regenerating tissues receive the metabolic support required for repair and functional recovery. Angiogenesis is particularly vital in contexts such as wound healing, myocardial repair, and bone regeneration [66]. VEGF plays a central role in creating new vascular networks, ensuring that regenerating tissues receive adequate oxygen and nutrients to support effective recovery [67]. Immunological training strategies that enhance angiogenesis, such as VEGF-boosting therapies, are particularly relevant in contexts where inadequate blood supply impairs healing. For example, in myocardial infarction and chronic wounds, targeted interventions that stimulate VEGF production have shown promise in accelerating vascularization and improving clinical outcomes [68]. Furthermore, therapies utilizing macrophage polarization to the M2 phenotype, which promotes pro-angiogenic activity, have demonstrated efficacy in preclinical models of ischemic injury [69]. These advances underscore the therapeutic potential of immune modulation in restoring vascular integrity and facilitating recovery.

- *The Immune System Modulates Local Stem Cell Niches*

Macrophages secrete transforming growth factor-beta (TGF-β) and insulin-like growth factor-1, which promote the proliferation and differentiation of resident stem cells. These factors facilitate tissue regeneration in multiple contexts, from skeletal muscle repair to neural regeneration. Additionally, the interplay between immune cells and local stem cell niches helps maintain a balance between repair and fibrosis, ensuring optimal functional recovery [70].

- *The Immune System Prevents Chronic Inflammation: The Role of Resolution Mediators*

While initiating inflammation is critical, the resolution phase is equally important for completing the healing process. Specialized pro-resolving mediators such as resolvins and protectins, derived from omega-3 fatty acids, play a central role in terminating inflammation and promoting tissue remodeling. Regulatory T cells (Tregs) and alternatively activated macrophages (M2 macrophages) contribute to anti-inflammatory signaling, promoting tissue remodeling and scar reduction. This resolution phase is critical for avoiding complications such as excessive fibrosis, which can impair functional recovery [71]. Immunological training can harness these molecules to accelerate the resolution phase, reducing the risk of chronic inflammation and fibrosis. For example, therapies that enhance the activity of Tregs or promote the M2 phenotype of macrophages are effective in creating an anti-inflammatory environment conducive to healing [72]. Or therapies targeting IL-10 and TGF-β can tilt the balance toward anti-inflammatory responses, optimizing the healing environment. Additionally, biomaterials that modulate immune responses are being developed to create pro-regenerative microenvironments. These advancements highlight the therapeutic potential of immune modulation in improving rehabilitation outcomes [73]. In conclusion, the immune system is a pivotal player in rehabilitation and tissue regeneration, orchestrating the processes of inflammation, angiogenesis, endogenous repair, and resolution. Advances in immunology have opened new ways for leveraging these mechanisms in therapeutic strategies, offering hope for enhanced recovery in a wide range of medical conditions. By understanding and harnessing the immune system’s complexity, researchers and clinicians can pave the way for more effective and holistic approaches to rehabilitation and regenerative medicine.

## 6. Immunological Training: Refining Biological Responses

As said, the immune system is a cornerstone of the body’s ability to heal and recover, performing a dual role as both a catalyst for tissue repair and a potential source of pathological complications when dysregulated. In this complex interplay, immunological training represents a cutting-edge approach that aims to harness the positive aspects of immune function while mitigating the risks of excessive inflammation or autoimmune complications [29]. Effective tissue regeneration depends on the immune system’s ability to balance pro-inflammatory and anti-inflammatory responses. An optimal immune response is characterized by a timely transition from the pro-inflammatory to the resolution phase, minimizing tissue damage while promoting repair. Dysregulation of this balance, whether through excessive inflammation or inadequate resolution, can result in chronic conditions such as fibrosis, delayed healing, and autoimmune diseases. Thus, understanding and manipulating this balance is a cornerstone of contemporary rehabilitation science [74]. Undoubtedly, immunological training may be an important mechanism for precision control of the immune system, as it involves interventions designed to enhance or suppress specific immune responses, depending on the clinical context. As such, immunological training represents a transformative approach in medical rehabilitation, redefining the role of the immune system as a carefully regulated driver of recovery [75]. At its core, this approach seeks to achieve precision control of immunological processes, tailoring their activity to meet the specific demands of tissue recovery. Key strategies in this regard include cytokine therapy, monoclonal antibodies, and adoptive immune cell transfer to target specific immune pathways to block pro-inflammatory signals or enhance reparative processes [41,65,66,71,73,74,76,77]. For example, therapies that enhance the activity of Tregs or promote the M2 phenotype of macrophages are effective in creating an anti-inflammatory environment conducive to healing [72]. Drugs such as infliximab, which inhibits TNF-α, have shown promise in reducing chronic inflammation in autoimmune diseases while supporting healing [78,79]. Proactive strategies, such as vaccines targeting specific immune pathways, are also being explored to prevent infections and enhance resilience during rehabilitation. Monoclonal antibodies that fine-tune immune responses, ensuring that they align with the body’s repair needs, are increasingly used [5,41,80]. In conclusion, immunological training is being increasingly applied across a spectrum of conditions, from acute injuries to chronic diseases. In conditions like rheumatoid arthritis, where the immune system plays a pathological role, therapies aim to suppress harmful immune responses while preserving reparative functions. Conversely, in cases of acute trauma or infection, immunological training focuses on amplifying the immune system’s ability to repair tissue and fight pathogens. The versatility of immunological approaches underscores their relevance to both rehabilitation and regenerative medicine [29,81]. This field is poised for significant advancements as new technologies enable more precise manipulation of the immune system. Bioinformatics and artificial intelligence are being used to identify novel immune targets, while nanotechnology facilitates the delivery of immunomodulatory agents with unprecedented accuracy [81]. Personalized medicine is also expected to play a crucial role, tailoring immunological interventions to the unique genetic and environmental factors influencing each patient’s recovery. As research continues to uncover the complexities of immune regulation, immunological training is set to become an integral component of holistic rehabilitation strategies, improving outcomes for patients worldwide [82].

## 7. The Role of the Microbiome

This section explores the microbiome’s profound influence on immune function, metabolic regulation, and systemic balance, positioning it as a cornerstone in modern rehabilitation strategies. The human microbiome, comprising trillions of microorganisms residing on and within the body, plays an essential role in maintaining homeostasis and promoting overall health [36,83]. As research into the microbiome advances, its critical connection to systemic health and rehabilitation outcomes becomes increasingly clear. The microbiome is intricately linked to metabolic processes, influencing everything from glucose regulation to energy expenditure. Bioactive metabolites interact with host metabolic pathways and play a crucial role in maintaining glucose and lipid homeostasis, regulating appetite, and preventing metabolic disorders [84,85]. In rehabilitation, targeting the microbiome to stabilize metabolic processes can have far-reaching benefits, particularly in patients with diabetes, obesity, or metabolic syndrome. Emerging therapies include fecal microbiome transplantation (FMT), which transfers healthy microbiota from a donor to a recipient to restore balance and improve metabolic outcomes [36]. These therapies exemplify how microbiome modulation can directly enhance rehabilitation strategies by addressing underlying metabolic dysfunctions. Box 2 exemplifies how the microbiome can influence regenerative processes.

Box 2 **The microbiome influences regenerative processes through several mechanisms.**

1. *Metabolic Modulation*: The gut microbiota can modulate metabolic pathways, which are crucial for tissue regen-eration. For example, certain bacterial candidates have been identified that potentially influence liver regeneration by modulating these pathways [86,87,88].
2. *Immune Modulation*: The gut microbiota serves as a pivotal regulator of immune system development and functionality [36,89]. During early life, exposure to diverse microbiota helps “train” the immune system, enabling it to distinguish between harmful pathogens and benign antigens. This process of immune education continues throughout life, with microbial metabolites influencing the differentiation of Tregs and modulating inflammatory responses [90].
3. *Neuro-regeneration*: The gut microbiota has been shown to impact the peripheral nervous system, affecting nerve injury and regeneration. This suggests a role for the microbiome in neuro-regenerative processes [38,89,91,92].
4. *Patterning and Development*: In some organisms, the microbiome can alter regenerative processes to influence developmental patterning outcomes, indicating a role in tissue and organ regeneration [93,94,95].
5. *Skin and Keratinocyte Function*: The skin microbiome influences host immunity and keratinocyte function, which are essential for skin regeneration and repair [96,97,98,99].
6. *Bone remodeling*: The gut microbiome may significantly influence bone metabolism, degenerative skeletal diseases and bone remodeling [100,101,102].

## 8. Prebiotics, Probiotics, and Postbiotics: Restoring Microbial Balance

Disruptions in the microbiome, known as dysbiosis, are linked to a wide range of health issues, including metabolic disorders, immune dysregulation, and chronic inflammation. Thus, restoring microbial balance is critical for maintaining systemic homeostasis [103,104]. In recent years, the emergence of evidence linking gut dysbiosis to a wide range of human diseases has sparked the development of microbiome-based therapeutic approaches [85,105]. Probiotics—live beneficial bacteria—and prebiotics—nutritional compounds that promote the growth of beneficial microbes—can enhance immune responses and reduce systemic inflammation [106,107]. Probiotics, indeed, help replenish beneficial bacterial populations, while prebiotics like inulin and fructooligosaccharides (FOS) feed these bacteria, supporting nutrient absorption, metabolic efficiency, and pathogen resistance. Although these approaches have shown promise in various disease contexts, their widespread use has been limited by numerous challenges, including the intestinal survival of orally administered probiotics or the risk of transferring potential pathogens to the new host by FMT [85,103]. In addition, as the existing microbial community influences the efficacy of these microbiome-based therapeutic strategies, the interindividual variability could be a limiting factor. However, postbiotic-based therapeutics can overcome these caveats. As microbial metabolites largely contribute to the beneficial effects of commensal microbes and their efficacy is less dependent on the composition of endogenous flora, their administration may be more universally applicable than targeting phylogeny [103]. Postbiotics, such as short-chain fatty acids (SCFAs), tryptophan (Trp) metabolites, bile acids (BAs), and polyamines (PAs)—in addition to others—have proven to directly influence host physiology by reducing oxidative stress, modulating immune responses, and promoting intestinal barrier integrity [108,109,110]. SCFAs such as acetate, butyrate, and propionate, produced by gut microbiota through the fermentation of fibers, act as signaling agents that influence inflammatory pathways, immune cell differentiation, and metabolic homeostasis. The implications of SCFAs extend beyond the gastrointestinal tract. Their ability to cross the gut barrier allows them to exert systemic effects, including modulation of pulmonary inflammation, improved insulin sensitivity, and neuroprotective functions. In the context of rehabilitation, SCFAs have been linked to enhanced recovery outcomes by mitigating inflammatory cascades and promoting a favorable immune response. Therapeutic strategies aimed at boosting SCFAs production through dietary prebiotics or targeted probiotic supplementation offer a promising avenue for integrating microbiota modulation into comprehensive rehabilitation frameworks [110,111].

Diverse biological roles for BAs have been recently discovered [112]. BAs are regulated by both the host and gut microbiota, serving as important host-microbiota messengers. Synthesized primarily in the liver, BAs are dynamically shaped by diverse bile acid metabolic enzymes, especially from gut microbiota. Manipulated by the gut microbiota and depending on the metabolic capacity of the bacterial community, the BAs pool is also influenced by external factors, such as antibiotics and diet. It is increasingly acknowledged how BA signaling networks are affected in distinct organs and how these networks affect energy [113] and skeletal muscle [114,115] homeostasis, likely impacting the outcome of regenerative interventions [116].

PAs (putrescine, spermidine, and spermine) are organic cations essential for normal cell growth as they modulate cell cycle progression and apoptosis, thus impacting senescence and longevity [117,118]. In general, PAs are involved in several important cellular processes and their disregulation can affect growth, aging and several diseases such as cancer, neurodegeneration and metabolic disorders. The peculiar activity of spermidine as an inducer of autophagy suggests the possibility of exploiting its use to enhance this cytoprotective mechanism to counteract the degenerative changes underlying either aging or degenerative diseases [119]. Studies have shown that nutritional supplementation with spermidine can reduce age-related pathology and increase life span in a number of organisms, including humans [39]. In addition, PAs regulate intestinal epithelial integrity by modulating the expression of various growth-related genes [120]. The ingested food is the major source of PAs in the upper parts of the intestine, while the gut microbiota is considered the main contributor to PA levels in the lower part of the intestine via de novo synthesis pathways and PAs uptake through specific transport systems [121]. PAs and PA-related enzymes have been implicated in bone development as global regulators of the transcriptional and translational activity of stem cells and pivotal transcription factors, suggesting their possible use as a tool to improve regenerative medicine strategies in orthopedics [122].

In recent years, microbial Trp metabolites, such as indole and derivatives, have also emerged as key metabolites [123,124]. Functioning as unique microbial molecules signaling via the aryl hydrocarbon receptor (AhR) [125], indole and derivatives thereof play a crucial role in maintaining health and immune homeostasis at mucosal surfaces [40,126]. Activation of AhR modulates cytokine production, enhances regulatory T-cell differentiation, and fosters anti-inflammatory environments conducive to tissue repair [108]. Studies have highlighted the therapeutic potential of indole-based postbiotics, particularly in enhancing systemic recovery mechanisms, sustaining embryonic stem cell self-renewal [127], axonal regeneration [128], myofiber formation [129], and supporting targeted rehabilitation strategies [130]. In pulmonary rehabilitation, indole-based postbiotics offer unique benefits through the gut-lung axis, a bidirectional relationship in which gut microbiota influence pulmonary health via immune and inflammatory pathways and viceversa [131,132]. Accumulating evidence also underscores the role of local microbial diversity in maintaining respiratory homeostasis as well as the role of dysbiosis in exacerbating lung inflammation, impaired recovery, and cancer [133]. Because of the multifactorial nature of many chronic human diseases, microbial metabolites capable of targeting multiple features of disease pathogenesis may offer the opportunity to greatly improve clinical outcomes. Considering the stability and the suitability for dose-dependent administration of postbiotics, they can be viewed as attractive therapeutic options. However, there are challenges associated with their administration, such as the rapid metabolism upon parenteral administration or the premature metabolism in the upper intestinal tract after oral administration. This necessitates the use of appropriate biopharmaceutical formulations designed to ensure controlled and targeted delivery of microbial metabolites, enhancing therapeutic efficacy while minimizing unwanted toxicities and preventing off-target effects [134]. By different mechanisms—including modulation of membrane potential of epithelial cells and alveolar macrophage functionality and reduction of cytokine-induced pulmonary inflammation—indole postbiotics support respiratory recovery. At the same time, indole derivatives can act as signaling molecules regulating microbial growth and virulence, thus contributing to microbial eubiosis and functioning [111,132,135]. Thus, by engaging specific molecular pathways, they may shape immune and microbial responses during recovery and rehabilitation. Integrating indole-based postbiotics into rehabilitation protocols holds promise for synergizing microbiome modulation with systemic and localized interventions. Together, probiotics, prebiotics, and postbiotics, by contributing to a stable internal environment, create a foundation for systemic recovery and resilience [136] (Table 2).

Altogether, microbiome-directed strategies, particularly when paired with regenerative medicine approaches, enhance outcomes by addressing microbial dysbiosis and leveraging microbiota-host interaction pathways. Undoubtedly, harnessing these mechanisms therapeutically could involve precision prebiotic formulations designed to enhance metabolite production or postbiotic approaches delivering bioactive microbial metabolites directly. The feasibility of respiratory administration, such as aerosolized formulations of indole derivatives, is thus an emerging strategy that aligns with personalized rehabilitation programs, optimizing recovery outcomes for diverse patient populations. Table 3 illustrates a potential therapeutic roadmap along this direction.

## 9. The Holistic Health Approach in Rehabilitation

As mentioned above, integrating microbiome management into rehabilitation strategies offers a holistic approach that addresses both physical and systemic health. By combining microbiome modulation with other rehabilitation strategies, such as regenerative medicine and immune training, a comprehensive recovery process can be achieved. This integration ensures that rehabilitation efforts target not only localized injuries but also systemic imbalances that could hinder full recovery. The expected result is a more robust, sustainable approach to healing in rehabilitation and other chronic conditions.

Taken as a whole, these considerations support the concept of the microbiome’s profound influence on immune function, metabolic regulation, and systemic homeostasis, making it an indispensable element of modern rehabilitation frameworks. By leveraging prebiotics, probiotics, postbiotics, and advanced microbiome-targeted therapies, clinicians can address the root causes of systemic dysfunction, creating a fertile environment for recovery.

## 10. Conclusions

**Figure 1:**
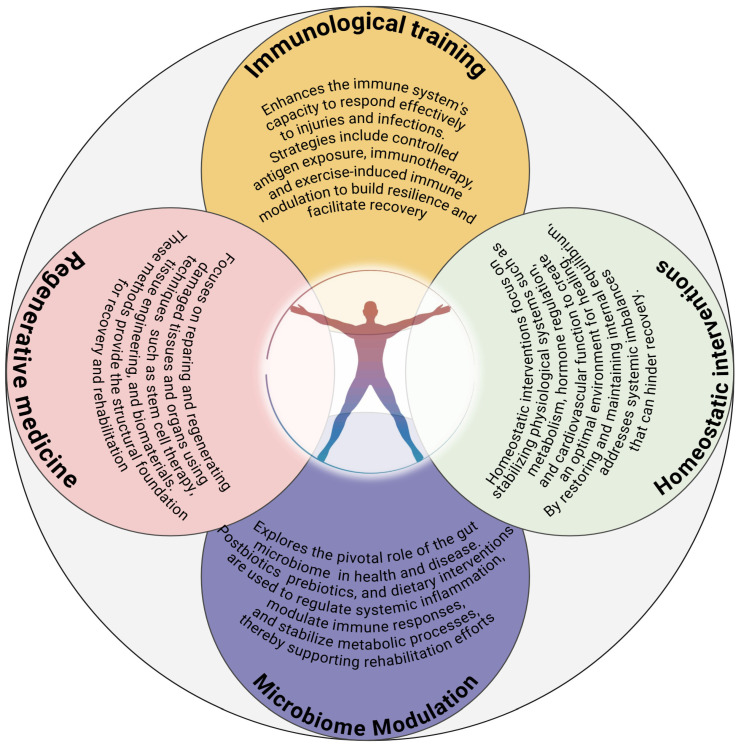
A multidimensional rehabilitation framework. This framework represents the interplay between key components essential to a comprehensive medical rehabilitation paradigm, ensuring internal homeostasis conducive to healing and optimal function—1. Regenerative Medicine: Focuses on repairing and regenerating damaged tissues and organs using techniques such as stem cell therapy, tissue engineering, and biomaterials. These methods provide the structural foundation for recovery and rehabilitation. 2. Homeostatic interventions: It focuses on restoring and maintaining internal equilibrium, addressing systemic imbalances that can hinder recovery. 3. Immunological Training: Enhances the immune system’s capacity to respond effectively to injuries and infections. Strategies include controlled antigen exposure, immunotherapy, and exercise-induced immune modulation to build resilience and facilitate recovery. 4. Microbiome Modulation: Explores the pivotal role of the gut microbiome in health and disease. These components converge in a holistic rehabilitation program (Integrated approach) addressing physical, immunological, and metabolic needs of the patient. Each component is integrated dynamically across the rehabilitation timeline. Personalized interventions are developed to optimize recovery, improve functional outcomes, and enhance quality of life.

The convergence of microbiome science and regenerative medicine offers unprecedented opportunities for enhancing rehabilitation outcomes. Probiotic and prebiotic therapies can complement regenerative strategies by creating a microenvironment conducive to cellular repair and tissue regeneration. Emerging research also suggests that combining microbiome-targeted interventions with stem cell therapies can synergistically improve outcomes. As the field advances, microbiome-based biomarkers are poised to become essential tools for predicting rehabilitation outcomes and tailoring interventions. Specific microbial signatures or metabolite profiles could guide therapeutic decisions, enabling clinicians to adopt a precision medicine approach. Furthermore, novel microbiome-targeted therapeutics, such as engineered probiotics or phage therapy, offer exciting prospects. Engineered probiotics could be designed to produce specific metabolites or modulate immune pathways, while phage therapy could selectively target pathogenic strains, restoring microbial balance without disrupting beneficial communities. These innovations hold significant promise for addressing the complex needs of patients undergoing rehabilitation. Translating microbiome research into clinical practice requires actionable strategies tailored to specific rehabilitation contexts. For pulmonary rehabilitation, dietary interventions rich in fermentable fibers, coupled with targeted probiotic supplementation, can enhance recovery by modulating gut-lung interactions. In chronic conditions such as diabetes or metabolic syndrome, microbiome-targeted therapies can stabilize systemic inflammation and improve metabolic control. Future protocols should also integrate patient-specific factors, such as genetic predispositions and baseline microbial diversity, to personalize interventions [146,147]. By leveraging microbiome science in conjunction with established rehabilitation methodologies, clinicians can achieve more comprehensive and sustainable recovery outcomes [133]. This integrative approach represents a paradigm shift in rehabilitation, focusing on systemic optimization rather than isolated interventions (Figure 1).

## Figures and Tables

**Table 1:** Comparative Table of Key Regenerative Medicine Techniques.

Technique	Description	Clinical Applications	Outcomes	References
Cell-based technologies	Use of stem cells to regenerate or repair damaged tissues.	Musculoskeletal injuries, neurodegenerative disorders, and cardiac repair.	Promotes tissue repair, reduces inflammation, and enhances functional recovery.	[47,48,49,50]
Tissue Engineering	Cell sources and growth factor, artificial and 3D-printed scaffolds.	Skin and vascular grafts, organ reconstruction, cartilage, and bone repair.	Enables anatomical restoration, improves structural integrity, and accelerates healing.	[51,52,53]
Gene delivery technologies	Introduction of genetic material to correct or modify cellular dysfunctions.	Genetic disorders, cancer, and immunodeficiencies.	Corrects genetic mutations, enhances targeted therapies, and improves cellular functionality.	[54,55,56]
Molecular therapies	Growth factors, small molecules, and cytokines to support tissue regeneration.	Autoimmune diseases, chronic inflammation, bone regeneration, transplant medicine.	Balances immune responses, reduces inflammation, enhances functional recovery.	[57,58,59]

**Table 2:** Roles of Probiotics, Prebiotics, and Postbiotics in Rehabilitation.

Component	Role	Mechanisms	Benefits	References
**Probiotics**	Live beneficial bacteria administered to restore microbial balance.	Compete with pathogens, produce bioactive compounds, and enhance immune cell activity.	Provide immune modulatory and biocompatibility effects in tissue engineering and improve wound healing in regenerative medicine.	[106,137,138,139]
**Prebiotics**	Nutritional compounds that promote the growth of beneficial bacteria.	Fermented by gut microbiota to produce bioactive metabolites.	Enhances gut microbiota diversity, supports nutrient absorption, and stabilizes homeostasis.	[140,141,142]
**Postbiotics**	Bioactive compounds produced by probiotics, such as SCFAs, BAs, PAs, indole derivatives or peptides.	Directly influence host physiology through anti-inflammatory and antioxidant effects, and promote gut barrier integrity.	Reduces oxidative stress, systemic inflammation, promotes tissue healing, and systemic homeostasis	[143,144,145]

**Table 3:** Therapeutic Roadmap: Steps for Integrating Microbiome Modulation into Personalized Rehabilitation Plans.

Step	Description	Actions	Expected Benefits
1. Baseline Assessment	Evaluate the patient’s microbiome profile and overall health status.	Conduct gut microbiota analysis, assess dietary habits, and identify dysbiosis or imbalances.	Personalized insights into microbiome health and targeted intervention planning.
2. Targeted Nutritional Plan	Design a dietary strategy to support microbial diversity and SCFA production.	Incorporate prebiotics (e.g., inulin, fructo-oligosaccharides; FOS) and fiber-rich foods into the patient’s diet.	Enhances gut microbiota diversity, supports metabolic homeostasis, and improves recovery.
3. Probiotic Supplementation	Introduce beneficial live bacteria tailored to individual needs.	Prescribe specific probiotic strains based on identified deficiencies (e.g., Lactobacillus, Bifidobacterium).	Restores microbial balance, reduces inflammation, and boosts immune resilience.
4. Postbiotic Integration	Incorporate bioactive metabolites produced by beneficial bacteria into the therapy plan.	Use SCFA supplements or postbiotic formulations to enhance systemic and localized recovery.	Strengthens gut barrier integrity, modulates immunity, and accelerates tissue healing.
5. Monitor and Adjust	Regularly assess microbiome-related health outcomes to refine the rehabilitation plan.	Perform follow-up microbiota analyses and adapt dietary or supplementation strategies.	Ensures sustained microbiome health and optimizes long-term rehabilitation outcomes.
6. Gut-Health Education	Empower patients with knowledge about maintaining a healthy microbiome.	Provide guidance on diet, lifestyle, and probiotic use to prevent dysbiosis.	Promotes long-term health resilience and prevents recurrence of imbalances.

## Data Availability

Data sharing is not applicable to this article as no datasets were generated or analyzed during the current study.

## Abbreviations

BAs	biliary acids
MSCs	mesenchymal stem cells
SCFAs	short-chain fatty acids
FMT	fecal microbiota transplantation
PAs	polyamines
TNF-α	tumor necrosis factor-alpha
VEGF	vascular endothelial growth factor
TGF-β	transforming growth factor-beta
Tregs	Regulatory T cells
M2	macrophages
IL	interleukin
Trp	tryptophan
AhR	aryl hydrocarbon receptor
FOS	fructo-oligosaccharides

## References

[1] Chua K.S.G., Kuah C.W.K. (2017). Innovating with Rehabilitation Technology in the Real World: Promises, Potentials, and Perspectives. Am. J. Phys. Med. Rehabil..

[2] Kannenberg A., Rupp R., Wurdeman S.R., Frossard L. (2024). Editorial: Advances in technology-assisted rehabilitation. Front. Rehabil. Sci..

[3] Kang Y.J., Zheng L. (2013). Rejuvenation: An integrated approach to regenerative medicine. Regen. Med. Res..

[4] Keever T. (2025). The Whole Well-Being Model: A Layered Framework for Thriving People, Systems, and Planet. Glob. Adv. Integr. Med. Health.

[5] Ten Brink T., Damanik F., Rotmans J.I., Moroni L. (2024). Unraveling and Harnessing the Immune Response at the Cell-Biomaterial Interface for Tissue Engineering Purposes. Adv. Healthc. Mater..

[6] Salgado A.J., Oliveira J.M., Martins A., Teixeira F.G., Silva N.A., Neves N.M., Sousa N., Reis R.L. (2013). Tissue engineering and regenerative medicine: Past, present, and future. Int. Rev. Neurobiol..

[7] Dzobo K., Thomford N.E., Senthebane D.A., Shipanga H., Rowe A., Dandara C., Pillay M., Motaung K. (2018). Advances in Regenerative Medicine and Tissue Engineering: Innovation and Transformation of Medicine. Stem Cells Int..

[8] Hammerhøj A., Chakravarti D., Sato T., Jensen K.B., Nielsen O.H. (2024). Organoids as regenerative medicine for inflammatory bowel disease. iScience.

[9] Zakrzewski J.L., van den Brink M.R., Hubbell J.A. (2014). Overcoming immunological barriers in regenerative medicine. Nat. Biotechnol..

[10] Petrus-Reurer S., Romano M., Howlett S., Jones J.L., Lombardi G., Saeb-Parsy K. (2021). Immunological considerations and challenges for regenerative cellular therapies. Commun. Biol..

[11] Shavandi A., Saeedi P., Gérard P., Jalalvandi E., Cannella D., Bekhit A.E.-D. (2020). The role of microbiota in tissue repair and regeneration. J. Tissue Eng. Regen. Med..

[12] Ogunrinola G.A., Oyewale J.O., Oshamika O.O., Olasehinde G.I. (2020). The Human Microbiome and Its Impacts on Health. Int. J. Microbiol..

[13] Afzaal M., Saeed F., Shah Y.A., Hussain M., Rabail R., Socol C.T., Hassoun A., Pateiro M., Lorenzo J.M., Rusu A.V. (2022). Human gut microbiota in health and disease: Unveiling the relationship. Front. Microbiol..

[14] Nunzi E., Pariano M., Costantini C., Garaci E., Puccetti P., Romani L. (2025). Host-microbe serotonin metabolism. Trends Endocrinol. Metab..

[15] Preethy S., Ranganathan N., Raghavan K., Dedeepiya V.D., Ikewaki N., Abraham S.J.K. (2022). Integrating the Synergy of the Gut Microbiome into Regenerative Medicine: Relevance to Neurological Disorders. J. Alzheimer’s Dis..

[16] Deng C., Aldali F., Luo H., Chen H. (2024). Regenerative rehabilitation: A novel multidisciplinary field to maximize patient outcomes. Med. Rev..

[17] Berthiaume F., Maguire T.J., Yarmush M.L. (2011). Tissue engineering and regenerative medicine: History, progress, and challenges. Annu. Rev. Chem. Biomol. Eng..

[18] de Jongh D., Massey E.K., Cronin A.J., Schermer M.H., Bunnik E.M., VANGUARD Consortium (2022). Early-Phase Clinical Trials of Bio-Artificial Organ Technology: A Systematic Review of Ethical Issues. Transplant. Int..

[19] Hunsberger J., Simon C., Zylberberg C., Ramamoorthy P., Tubon T., Bedi R., Gielen K., Hansen C., Fischer L., Johnson J. (2020). Improving patient outcomes with regenerative medicine: How the Regenerative Medicine Manufacturing Society plans to move the needle forward in cell manufacturing, standards, 3D bioprinting, artificial intelligence-enabled automation, education, and training. STEM CELLS Transl. Med..

[20] Makuku R., Werthel J.D., Zanjani L.O., Nabian M.H., Tantuoyir M.M. (2022). New frontiers of tendon augmentation technology in tissue engineering and regenerative medicine: A concise literature review. J. Int. Med. Res..

[21] Saini G., Segaran N., Mayer J.L., Saini A., Albadawi H., Oklu R. (2021). Applications of 3D Bioprinting in Tissue Engineering and Regenerative Medicine. J. Clin. Med..

[22] Feng J.N., Jin T. (2022). Hormones that are involved in metabolic homeostasis: Overview of the past century and future perspectives. Obes. Med..

[23] Liu H., Wang S., Wang J., Guo X., Song Y., Fu K., Gao Z., Liu D., He W., Yang L.L. (2025). Energy metabolism in health and diseases. Signal Transduct. Target. Ther..

[24] Messonnier L.A. (2023). Physical Exercise or Activity and Energy Balance or Metabolism in the Context of Health and Diseases. Nutrients.

[25] Tao Z., Cheng Z. (2023). Hormonal regulation of metabolism—Recent lessons learned from insulin and estrogen. Clin. Sci..

[26] Julier Z., Park A.J., Briquez P.S., Martino M.M. (2017). Promoting tissue regeneration by modulating the immune system. Acta Biomater..

[27] Bindu S., Dandapat S., Manikandan R., Dinesh M., Subbaiyan A., Mani P., Dhawan M., Tiwari R., Bilal M., Emran T.B. (2022). Prophylactic and therapeutic insights into trained immunity: A renewed concept of innate immune memory. Human. Vaccines Immunother..

[28] Li F., Ouyang J., Chen Z., Zhou Z., Milon Essola J., Ali B., Wu X., Zhu M., Guo W., Liang X.J. (2024). Nanomedicine for T-Cell Mediated Immunotherapy. Adv. Mater..

[29] Netea M.G., Domínguez-Andrés J., Barreiro L.B., Chavakis T., Divangahi M., Fuchs E., Joosten L.A.B., van der Meer J.W.M., Mhlanga M.M., Mulder W.J.M. (2020). Defining trained immunity and its role in health and disease. Nat. Rev. Immunol..

[30] de Sire A., de Sire R., Curci C., Castiglione F., Wahli W. (2022). Role of dietary supplements and probiotics in modulating microbiota and bone health: The gut-bone axis. Cells.

[31] Liu Y., Wang J., Wu C. (2022). Modulation of Gut Microbiota and Immune System by Probiotics, Pre-biotics, and Post-biotics. Front. Nutr..

[32] Martyniak A., Medyńska-Przęczek A., Wędrychowicz A., Skoczeń S., Tomasik P.J. (2021). Prebiotics, Probiotics, Synbiotics, Paraprobiotics and Postbiotic Compounds in IBD. Biomolecules.

[33] Zelante T., Iannitti R.G., Cunha C., De Luca A., Giovannini G., Pieraccini G., Zecchi R., D’Angelo C., Massi-Benedetti C., Fallarino F. (2013). Tryptophan catabolites from microbiota engage aryl hydrocarbon receptor and balance mucosal reactivity via interleukin-22. Immunity.

[34] Hou K., Wu Z.X., Chen X.Y., Wang J.Q., Zhang D., Xiao C., Zhu D., Koya J.B., Wei L., Li J. (2022). Microbiota in health and diseases. Signal Transduct. Target. Ther..

[35] Mazziotta C., Tognon M., Martini F., Torreggiani E., Rotondo J.C. (2023). Probiotics Mechanism of Action on Immune Cells and Beneficial Effects on Human Health. Cells.

[36] Rooks M.G., Garrett W.S. (2016). Gut microbiota, metabolites and host immunity. Nat. Rev. Immunol..

[37] Williams K.L., Enslow R., Suresh S., Beaton C., Hodge M., Brooks A.E. (2023). Using the Microbiome as a Regenerative Medicine Strategy for Autoimmune Diseases. Biomedicines.

[38] Belkaid Y., Hand T.W. (2014). Role of the microbiota in immunity and inflammation. Cell.

[39] López-Otín C., Kroemer G. (2021). Hallmarks of Health. Cell.

[40] Zelante T., Puccetti M., Giovagnoli S., Romani L. (2021). Regulation of host physiology and immunity by microbial indole-3-aldehyde. Curr. Opin. Immunol..

[41] Aurora A.B., Olson E.N. (2014). Immune modulation of stem cells and regeneration. Cell Stem Cell.

[42] Han X., Liao R., Li X., Zhang C., Huo S., Qin L., Xiong Y., He T., Xiao G., Zhang T. (2025). Mesenchymal stem cells in treating human diseases: Molecular mechanisms and clinical studies. Signal Transduct. Target. Ther..

[43] Naik S., Larsen S.B., Cowley C.J., Fuchs E. (2018). Two to Tango: Dialog between Immunity and Stem Cells in Health and Disease. Cell.

[44] Velikic G., Maric D.M., Maric D.L., Supic G., Puletic M., Dulic O., Vojvodic D. (2024). Harnessing the Stem Cell Niche in Regenerative Medicine: Innovative Avenue to Combat Neurodegenerative Diseases. Int. J. Mol. Sci..

[45] Yu Y., Wang Q., Wang C., Shang L. (2021). Living Materials for Regenerative Medicine. Eng. Regen..

[46] Arsiwala A., Desai P., Patravale V. (2014). Recent advances in micro/nanoscale biomedical implants. J. Control Release.

[47] Guillamat-Prats R. (2021). The Role of MSC in Wound Healing, Scarring and Regeneration. Cells.

[48] Rajabzadeh N., Fathi E., Farahzadi R. (2019). Stem cell-based regenerative medicine. Stem Cell Investig..

[49] Takata N., Eiraku M. (2018). Stem cells and genome editing: Approaches to tissue regeneration and regenerative medicine. J. Hum. Genet..

[50] Wang Y., Gao T., Wang B. (2023). Application of mesenchymal stem cells for anti-senescence and clinical challenges. Stem Cell Res. Ther..

[51] Patel K.D., Lamarra K.A., Sawadkar P., Ludwig A., Perriman A.W. (2025). Silk Fibroin/GelMA-Based Hydrogels as a Platform for Tissue Adhesives and Tissue Engineering. ACS Biomater. Sci. Eng..

[52] Sharma P., Kumar P., Sharma R., Bhatt V.D., Dhot P.S. (2019). Tissue Engineering; Current Status & Futuristic Scope. J. Med. Life.

[53] Zhang J., Wehrle E., Rubert M., Muller R. (2021). 3D Bioprinting of Human Tissues: Biofabrication, Bioinks, and Bioreactors. Int. J. Mol. Sci..

[54] Hosseinkhani H., Domb A.J., Sharifzadeh G., Nahum V. (2023). Gene Therapy for Regenerative Medicine. Pharmaceutics.

[55] Saraf A., Mikos A.G. (2006). Gene delivery strategies for cartilage tissue engineering. Adv. Drug Deliv. Rev..

[56] Wu Q.X., De Isla N., Zhang L. (2025). Biomaterial-Based Nucleic Acid Delivery Systems for In Situ Tissue Engineering and Regenerative Medicine. Int. J. Mol. Sci..

[57] Chua C.Y.X., Jiang A.Y., Eufrasio-da-Silva T., Dolatshahi-Pirouz A., Langer R., Orive G., Grattoni A. (2023). Emerging immunomodulatory strategies for cell therapeutics. Trends Biotechnol..

[58] Moon S., Hong J., Go S., Kim B.S. (2023). Immunomodulation for Tissue Repair and Regeneration. Tissue Eng. Regen. Med..

[59] Oliveira E.R., Nie L., Podstawczyk D., Allahbakhsh A., Ratnayake J., Brasil D.L., Shavandi A. (2021). Advances in Growth Factor Delivery for Bone Tissue Engineering. Int. J. Mol. Sci..

[60] Kotas M.E., Medzhitov R. (2015). Homeostasis, inflammation, and disease susceptibility. Cell.

[61] Modell H., Cliff W., Michael J., McFarland J., Wenderoth M.P., Wright A. (2015). A physiologist’s view of homeostasis. Adv. Physiol. Educ..

[62] Wang S., Qin L. (2022). Homeostatic medicine: A strategy for exploring health and disease. Curr. Med..

[63] Nahrendorf M., Ginhoux F., Swirski F.K. (2025). Immune system influence on physiology. Science.

[64] Zhao H., Wu L., Yan G., Chen Y., Zhou M., Wu Y., Li Y. (2021). Inflammation and tumor progression: Signaling pathways and targeted intervention. Signal Transduct. Target. Ther..

[65] Medzhitov R. (2008). Origin and physiological roles of inflammation. Nature.

[66] Tonnesen M.G., Feng X., Clark R.A. (2000). Angiogenesis in wound healing. J. Investig. Dermatol. Symp. Proc..

[67] Carmeliet P., Jain R.K. (2011). Molecular mechanisms and clinical applications of angiogenesis. Nature.

[68] Chu H., Wang Y. (2012). Therapeutic angiogenesis: Controlled delivery of angiogenic factors. Ther. Deliv..

[69] Li W., Xu Z., Zou B., Yang D., Lu Y., Zhang X., Zhang C., Li Y., Zhu C. (2025). Macrophage regulation in vascularization upon regeneration and repair of tissue injury and engineered organ transplantation. Fundam. Res..

[70] Wynn T.A., Barron L. (2010). Macrophages: Master regulators of inflammation and fibrosis. Semin. Liver Dis..

[71] Serhan C.N. (2014). Pro-resolving lipid mediators are leads for resolution physiology. Nature.

[72] Clark M., Suur B.E., Sotak M., Borgeson E. (2024). Attenuation of adipose tissue inflammation by pro-resolving lipid mediators. Curr. Opin. Endocr. Metab. Res..

[73] Mantovani A., Biswas S.K., Galdiero M.R., Sica A., Locati M. (2013). Macrophage plasticity and polarization in tissue repair and remodelling. J. Pathol..

[74] Nathan C., Ding A. (2010). Nonresolving inflammation. Cell.

[75] Salauddin M., Nath S.K., Saha S., Zheng Q., Zheng C., Hossain M.G. (2024). Trained immunity: A revolutionary immunotherapeutic approach. Anim. Dis..

[76] McInnes I.B., Gravallese E.M. (2021). Immune-mediated inflammatory disease therapeutics: Past, present and future. Nat. Rev. Immunol..

[77] Song Y., Li J., Wu Y. (2024). Evolving understanding of autoimmune mechanisms and new therapeutic strategies of autoimmune disorders. Signal Transduct. Target. Ther..

[78] Livia C., Inglis S., Crespo-Diaz R., Rizzo S., Mahlberg R., Bagwell M., Hillestad M., Yamada S., Meenakshi Siddharthan D.V., Singh R.D. (2024). Infliximab Limits Injury in Myocardial Infarction. J. Am. Heart Assoc..

[79] Stratos I., Behrendt A.K., Anselm C., Gonzalez A., Mittlmeier T., Vollmar B. (2022). Inhibition of TNF-alpha Restores Muscle Force, Inhibits Inflammation, and Reduces Apoptosis of Traumatized Skeletal Muscles. Cells.

[80] Zarubova J., Hasani-Sadrabadi M.M., Ardehali R., Li S. (2022). Immunoengineering strategies to enhance vascularization and tissue regeneration. Adv. Drug Deliv. Rev..

[81] Vuscan P., Kischkel B., Joosten L.A.B., Netea M.G. (2024). Trained immunity: General and emerging concepts. Immunol. Rev..

[82] Clemente-Suárez V.J., Martín-Rodríguez A., Redondo-Flórez L., López-Mora C., Yáñez-Sepúlveda R., Tornero-Aguilera J.F. (2023). New Insights and Potential Therapeutic Interventions in Metabolic Diseases. Int. J. Mol. Sci..

[83] Chen Y., Fang J.-Y. (2025). The role of colonic microbiota amino acid metabolism in gut health regulation. Cell Insight.

[84] Ma T., Shen X., Shi X., Sakandar H.A., Quan K., Li Y., Jin H., Kwok L.-Y., Zhang H., Sun Z. (2023). Targeting gut microbiota and metabolism as the major probiotic mechanism—An evidence-based review. Trends Food Sci. Technol..

[85] Porcari S., Ng S.C., Zitvogel L., Sokol H., Weersma R.K., Elinav E., Gasbarrini A., Cammarota G., Tilg H., Ianiro G. (2025). The microbiome for clinicians. Cell.

[86] Liu H.X., Keane R., Sheng L., Wan Y.J. (2015). Implications of microbiota and bile acid in liver injury and regeneration. J. Hepatol..

[87] Zheng Z., Wang B. (2021). The gut-liver axis in health and disease: The role of gut microbiota-derived signals in liver injury and regeneration. Front. Immunol..

[88] Yin Y., Sichler A., Ecker J., Laschinger M., Liebisch G., Höring M., Basic M., Bleich A., Zhang X.J., Kübelsbeck L. (2023). Gut microbiota promote liver regeneration through hepatic membrane phospholipid biosynthesis. J. Hepatol..

[89] Zheng D., Liwinski T., Elinav E. (2020). Interaction between microbiota and immunity in health and disease. Cell Res..

[90] Kim C.H. (2023). Complex regulatory effects of gut microbial short-chain fatty acids on immune tolerance and autoimmunity. Cell Mol. Immunol..

[91] Liu C., Yang S.Y., Wang L., Zhou F. (2022). The gut microbiome: Implications for neurogenesis and neurological diseases. Neural Regen. Res..

[92] Park J.C., Chang L., Kwon H.K., Im S.H. (2025). Beyond the gut: Decoding the gut-immune-brain axis in health and disease. Cell Mol. Immunol..

[93] Williams K.B., Bischof J., Lee F.J., Miller K.A., LaPalme J.V., Wolfe B.E., Levin M. (2020). Regulation of axial and head patterning during planarian regeneration by a commensal bacterium. Mech. Dev..

[94] Tung A., Levin M. (2020). Extra-genomic instructive influences in morphogenesis: A review of external signals that regulate growth and form. Dev. Biol..

[95] Tran S., Stephanie Chen Y.F., Xu D., Ison M., Yang L. (2023). Microbial pattern recognition suppresses de novo organogenesis. Development.

[96] Kobayashi T., Naik S., Nagao K. (2019). Choreographing Immunity in the Skin Epithelial Barrier. Immunity.

[97] Piipponen M., Li D., Landén N.X. (2020). The Immune Functions of Keratinocytes in Skin Wound Healing. Int. J. Mol. Sci..

[98] Wang G., Sweren E., Liu H., Wier E., Alphonse M.P., Chen R., Islam N., Li A., Xue Y., Chen J. (2021). Bacteria induce skin regeneration via IL-1β signaling. Cell Host Microbe.

[99] Uberoi A., Bartow-McKenney C., Zheng Q., Flowers L., Campbell A., Knight S.A.B., Chan N., Wei M., Lovins V., Bugayev J. (2021). Commensal microbiota regulates skin barrier function and repair via signaling through the aryl hydrocarbon receptor. Cell Host Microbe.

[100] Chen Y., Wang X., Zhang C., Liu Z., Li C., Ren Z. (2022). Gut Microbiota and Bone Diseases: A Growing Partnership. Front. Microbiol..

[101] Filosa A., Sawamiphak S. (2023). Heart development and regeneration-a multi-organ effort. FEBS J..

[102] Lyu Z., Hu Y., Guo Y., Liu D. (2023). Modulation of bone remodeling by the gut microbiota: A new therapy for osteoporosis. Bone Res..

[103] Suez J., Elinav E. (2017). The path towards microbiome-based metabolite treatment. Nat. Microbiol..

[104] Ratiner K., Ciocan D., Abdeen S.K., Elinav E. (2024). Utilization of the microbiome in personalized medicine. Nat. Rev. Microbiol..

[105] Zmora N., Zeevi D., Korem T., Segal E., Elinav E. (2016). Taking it Personally: Personalized Utilization of the Human Microbiome in Health and Disease. Cell Host Microbe.

[106] Suez J., Zmora N., Segal E., Elinav E. (2019). The pros, cons, and many unknowns of probiotics. Nat. Med..

[107] Virk M.S., Virk M.A., He Y., Tufail T., Gul M., Qayum A., Rehman A., Rashid A., Ekumah J.N., Han X. (2024). The Anti-Inflammatory and Curative Exponent of Probiotics: A Comprehensive and Authentic Ingredient for the Sustained Functioning of Major Human Organs. Nutrients.

[108] Agus A., Clément K., Sokol H. (2021). Gut microbiota-derived metabolites as central regulators in metabolic disorders. Gut.

[109] Takeuchi T., Nakanishi Y., Ohno H. (2024). Microbial Metabolites and Gut Immunology. Annu. Rev. Immunol..

[110] Du Y., He C., An Y., Huang Y., Zhang H., Fu W., Wang M., Shan Z., Xie J., Yang Y. (2024). The Role of Short Chain Fatty Acids in Inflammation and Body Health. Int. J. Mol. Sci..

[111] Kumar P., Lee J.H., Lee J. (2021). Diverse roles of microbial indole compounds in eukaryotic systems. Biol. Rev. Camb. Philos. Soc..

[112] Collins S.L., Stine J.G., Bisanz J.E., Okafor C.D., Patterson A.D. (2023). Bile acids and the gut microbiota: Metabolic interactions and impacts on disease. Nat. Rev. Microbiol..

[113] Garruti G., Baj J., Cignarelli A., Perrini S., Giorgino F. (2023). Hepatokines, bile acids and ketone bodies are novel Hormones regulating energy homeostasis. Front. Endocrinol..

[114] Jia F., Liu X., Liu Y. (2025). Bile acid signaling in skeletal muscle homeostasis: From molecular mechanisms to clinical applications. Front. Endocrinol..

[115] Marullo A.L., O’Halloran K.D. (2023). Microbes, metabolites and muscle: Is the gut-muscle axis a plausible therapeutic target in Duchenne muscular dystrophy?. Exp. Physiol..

[116] Ma H., Wang K., Jiang C. (2025). Microbiota-derived bile acid metabolic enzymes and their impacts on host health. Cell Insight.

[117] Stolarska E., Paluch-Lubawa E., Grabsztunowicz M., Kumar Tanwar U., Arasimowicz-Jelonek M., Phanstiel O., Mattoo A.K., Sobieszczuk-Nowicka E. (2023). Polyamines as Universal Bioregulators Across Kingdoms and Their role in Cellular Longevity and Death. Crit. Rev. Plant Sci..

[118] Igarashi K., Kashiwagi K. (2010). Modulation of cellular function by polyamines. Int. J. Biochem. Cell Biol..

[119] Satarker S., Wilson J., Kolathur K.K., Mudgal J., Lewis S.A., Arora D., Nampoothiri M. (2024). Spermidine as an epigenetic regulator of autophagy in neurodegenerative disorders. Eur. J. Pharmacol..

[120] Timmons J., Chang E.T., Wang J.Y., Rao J.N. (2012). Polyamines and Gut Mucosal Homeostasis. J. Gastrointest. Dig. Syst..

[121] Tofalo R., Cocchi S., Suzzi G. (2019). Polyamines and Gut Microbiota. Front. Nutr..

[122] Borzi R.M., Guidotti S., Minguzzi M., Facchini A., Platano D., Trisolino G., Filardo G., Cetrullo S., D’Adamo S., Stefanelli C. (2014). Polyamine delivery as a tool to modulate stem cell differentiation in skeletal tissue engineering. Amino Acids.

[123] Agus A., Planchais J., Sokol H. (2018). Gut Microbiota Regulation of Tryptophan Metabolism in Health and Disease. Cell Host Microbe.

[124] Roager H.M., Licht T.R. (2018). Microbial tryptophan catabolites in health and disease. Nat. Commun..

[125] Hubbard T.D., Murray I.A., Bisson W.H., Lahoti T.S., Gowda K., Amin S.G., Patterson A.D., Perdew G.H. (2015). Adaptation of the human aryl hydrocarbon receptor to sense microbiota-derived indoles. Sci. Rep..

[126] Li S. (2023). Modulation of immunity by tryptophan microbial metabolites. Front. Nutr..

[127] Miyabayashi T., Yamamoto M., Sato A., Sakano S., Takahashi Y. (2008). Indole derivatives sustain embryonic stem cell self-renewal in long-term culture. Biosci. Biotechnol. Biochem..

[128] Borowski A.N., Vuong H.E. (2022). Crushing it: Indole-3 propionate promotes axonal regeneration in mice. Cell Host Microbe.

[129] Kong Y., Wang Q., Wang J., Qiu X., Yang Y., Liu J., Yang F., Qi R. (2025). Indole-3-propionic acid enhances glycolytic myofiber formation in piglets through PI3K-mTOR activation and gut microbiota-driven tryptophan metabolic alteration. Anim. Nutr..

[130] Hijova E. (2024). Postbiotics as Metabolites and Their Biotherapeutic Potential. Int. J. Mol. Sci..

[131] Melo-Dias S., Cabral M., Furtado A., Souto-Miranda S., Mendes M.A., Cravo J., Almeida C.R., Marques A., Sousa A. (2023). Responsiveness to pulmonary rehabilitation in COPD is associated with changes in microbiota. Respir. Res..

[132] Ye X., Li H., Anjum K., Zhong X., Miao S., Zheng G., Liu W., Li L. (2022). Dual Role of Indoles Derived from Intestinal Microbiota on Human Health. Front. Immunol..

[133] Kim Y.C., Sohn K.H., Kang H.R. (2024). Gut microbiota dysbiosis and its impact on asthma and other lung diseases: Potential therapeutic approaches. Korean J. Intern. Med..

[134] Puccetti M., Pariano M., Wojtylo P., Schoubben A., Giovagnoli S., Ricci M. (2023). Turning Microbial AhR Agonists into Therapeutic Agents via Drug Delivery Systems. Pharmaceutics.

[135] Kumar A., Sperandio V. (2019). Indole Signaling at the Host-Microbiota-Pathogen Interface. mBio.

[136] Mafe A.N., Iruoghene Edo G., Akpoghelie P.O., Gaaz T.S., Yousif E., Zainulabdeen K., Isoje E.F., Igbuku U.A., Opiti R.A., Garba Y. (2025). Probiotics and Food Bioactives: Unraveling Their Impact on Gut Microbiome, Inflammation, and Metabolic Health. Probiotics Antimicrob. Proteins.

[137] Aponte M., Murru N., Shoukat M. (2020). Therapeutic, Prophylactic, and Functional Use of Probiotics: A Current Perspective. Front. Microbiol..

[138] Bodke H., Jogdand S. (2022). Role of Probiotics in Human Health. Cureus.

[139] Golchin A., Ranjbarvan P., Parviz S., Shokati A., Naderi R., Rasmi Y., Kiani S., Moradi F., Heidari F., Saltanatpour Z. (2023). The role of probiotics in tissue engineering and regenerative medicine. Regen. Med..

[140] Choque Delgado G.T., Tamashiro W. (2018). Role of prebiotics in regulation of microbiota and prevention of obesity. Food Res. Int..

[141] Porcari S., Fusco W., Spivak I., Fiorani M., Gasbarrini A., Elinav E., Cammarota G., Ianiro G. (2024). Fine-tuning the gut ecosystem: The current landscape and outlook of artificial microbiome therapeutics. Lancet Gastroenterol. Hepatol..

[142] Yoo S., Jung S.C., Kwak K., Kim J.S. (2024). The Role of Prebiotics in Modulating Gut Microbiota: Implications for Human Health. Int. J. Mol. Sci..

[143] Lin W., Kuo Y.-W., Chen C.-W., Hsu Y.-C., Huang Y.-F., Hsu C.-H., Lin J.-H., Lin C.-H., Lin C.-C., Yi T.-H. (2022). The Function of Mixed Postbiotic PE0401 in Improving Intestinal Health via Elevating Anti-inflammation, Anti-oxidation, Epithelial Tight Junction Gene Expression and Promoting Beneficial Bacteria Growth. J. Pure Appl. Microbiol..

[144] Dinic M., Burgess J.L., Lukic J., Catanuto P., Radojevic D., Marjanovic J., Verpile R., Thaller S.R., Gonzalez T., Golic N. (2024). Postbiotic lactobacilli induce cutaneous antimicrobial response and restore the barrier to inhibit the intracellular invasion of Staphylococcus aureus in vitro and ex vivo. FASEB J..

[145] Spivak I., Fluhr L., Elinav E. (2022). Local and systemic effects of microbiome-derived metabolites. EMBO Rep..

[146] Chotirmall S.H., Bogaert D., Chalmers J.D., Cox M.J., Hansbro P.M., Huang Y.J., Molyneaux P.L., O’Dwyer D.N., Pragman A.A., Rogers G.B. (2022). Therapeutic Targeting of the Respiratory Microbiome. Am. J. Respir. Crit. Care Med..

[147] Druszczynska M., Sadowska B., Kulesza J., Gasienica-Gliwa N., Kulesza E., Fol M. (2024). The Intriguing Connection Between the Gut and Lung Microbiomes. Pathogens.

